# Improving cancer detection through computer-aided diagnosis: A comprehensive analysis of nonlinear and texture features in breast thermograms

**DOI:** 10.1371/journal.pone.0322934

**Published:** 2025-05-29

**Authors:** Hamed Khodadadi, Shima Nazem

**Affiliations:** 1 Department of Electrical Engineering, Khomeinishahr Branch, Islamic Azad University, Isfahan, Iran; 2 Department of Molecular Genetics, Faculty of Biological Sciences, Tarbiat Modares University, Tehran, Iran; Prince Mohammad Bin Fahd University, SAUDI ARABIA

## Abstract

Breast cancer is a significant health issue for women, characterized by its high rates of mortality and sickness. However, its early detection is crucial for improving patient outcomes. Thermography, which measures temperature variations between healthy and cancerous tissues, offers a promising approach for early diagnosis. This study proposes a novel method for analyzing breast thermograms. The method segments suspicious masses, extracts relevant features, and classifies them as benign or malignant. While the chaotic indices, including Lyapunov Exponent (LE), Fractal Dimension (FD), Kolmogorov–Sinai Entropy (KSE), and Correlation Dimension (CD), are employed for nonlinear analysis, the Gray-Level Co-occurrence Matrix (GLCM) method utilized for extracting the texture features. The effectiveness of the proposed approach is enhanced by integrating texture and complexity features. Besides, to optimize feature selection and reduce redundancy, a metaheuristic optimization technique called Non-Dominated Sorting Genetic Algorithm (NSGA III) is applied. The proposed method utilizes various machine learning algorithms, including Support Vector Machine (SVM), K-Nearest Neighbors (KNN), Linear Discriminant Analysis (LDA), Pattern recognition Network (Pat net), and Fitting neural Network (Fit net), for classification. ten-fold cross-validation ensures robust performance evaluation. The achieved accuracy of 98.65%, emphasizes the superior performance of the proposed method in thermograms breast cancer diagnosis.

## 1. Introduction

### 1.1. Background and motivations

Breast cancer has developed as a notable global health issue, standing as one of the primary contributors to global mortality rates. According to the Global Cancer Observatory (GLOBOCON) in 2020, female breast cancer had an estimated incidence rate of 11.7%, resulting in 2.3 million new cases worldwide [1]. With a mortality rate of approximately 13.6%, this cancer type accounts for 1 in 6 deaths related to cancer, underscoring the crucial need for timely detection and treatment to enhance survival rates. Besides, the World Health Organization (WHO) emphasizes breast cancer’s global impact, with 2.3 million new cases and 685,000 deaths in 2020. Efforts to enhance diagnostic capabilities are crucial especially in the regions with high breast cancer rates. Advancements in breast cancer detection, including professional visual examinations to detect palpable lumps indicating cancerous tissue, are vital for accurate assessment and treatment planning (WHO, 2020) [2,3].

Various imaging techniques such as mammography, sonography, Magnetic Resonance Imaging (MRI), Single Photon Emission Computed Tomography (SPECT), Positron Emission Tomography (PET), optical, and microwave imaging are commonly utilized for breast cancer diagnosis. Despite being the standard method, mammography has limitations like radiation exposure, patient discomfort, and difficulty in imaging dense tissues [4]. Consequently, current research aims to enhance detection rates using existing methods and explore new diagnostic options. Additionally, to aid radiologists in improving mammography screening accuracy, Computer-Aided Diagnostic (CAD) software has been developed and used clinically. However, disappointing data indicates that CAD methods applying mammograms did not enhance diagnostic accuracy significantly [5]. Therefore, there is an urgent need for alternative detection methods in breast cancer diagnosis.

**Fig. 1:**
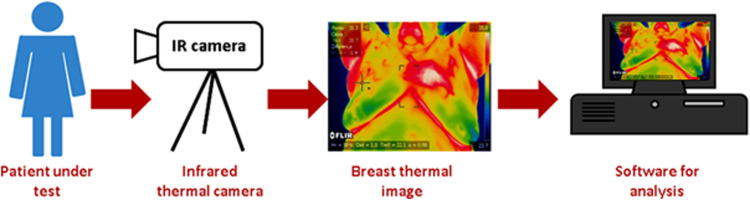
The procedure of acquiring breast thermograms [12].

Recent advancements in infrared camera technology have generated clinical interest in thermal imaging, known as thermography, involves measuring the heat emitted by different parts of the human body. A patient’s thermogram provides a visual representation of heat distribution throughout the body. Tumors exhibit higher temperatures than surrounding normal tissue due to their elevated metabolic rate and increased vascular angiogenesis. Infrared imaging, a non-invasive method, is utilized as a diagnostic tool for various diseases such as cancer, diabetes, and hypertension [6]. Consequently, breast cancer tissue appears as a distinct focal point in infrared images. Modern infrared cameras are precisely calibrated, and there exist well-established standards for thermography. Breast thermography offers several advantages, including non-invasiveness, absence of radiation, quickness, painlessness, and cost-effectiveness [7–10]. Moreover, thermography is suitable for women of all ages, including pregnant women and those with dense breast tissue [11]. Fig 1 illustrates the process of acquiring breast thermographic images.

### 1.2. Literature review

Most of the research performed on breast cancer diagnosis using thermograms relies on the publicly accessible Database for Mastology Research with Infrared Image (DMR-IR) database [13]. Consequently, this paper will provide an overview of studies that have utilized this dataset. Considering the typical CAD system design for breast cancer detection involves preprocessing, segmentation, feature extraction, selection, and classification, a review of existing studies in these areas can be conducted [14,15].

An extended hidden Markov models, Bayesian networks, and random forest are employed in [16] to optimize breast segmentation techniques. However, their proposed method is limited to initial stages in automated or semi-automated systems and their algorithm requires enhancement for real-time applications. An automatic segmentation approach for extracting the ROI from breast thermograms was presented in [17]. The method relied on statistical and texture features extracted from normal and abnormal breasts. Besides, the dataset used in the study was limited and resulted in the mistakenly removal of some lower breast regions. An enhanced segmentation method was proposed in [18] via combining Neutrosophic sets with an optimized fast Fuzzy c-mean algorithm. Subsequently, different kernel functions of SVM were applied to discriminate between normal and abnormal breast tissue. The reported accuracy, recall, and precision rates were 92.06%, 96.55%, and 87.50%, respectively.

Besides, the application of various machine learning techniques, including SVM, KNN, Random Forest (RF), and Decision Tree (DT), for classifying breast images and detecting cancer investigated in [19]. Scale-Invariant Feature Transform (SIFT) and Speeded Up Robust Features (SURF) feature extraction methods were employed to characterize the image data. A comparative analysis of these machine learning models was conducted to evaluate their performance in cancer detection. Accurate segmentation of suspicious regions in thermal breast images which is crucial for breast cancer detection is considered in [20]. This study proposes a novel active contour model for this task that combines initial region localization, a refined energy function, and an optimized stopping criterion to improve segmentation accuracy and speed. Evaluation on standard datasets demonstrates superior performance compared to existing approaches. Furthermore, the segmented regions are effectively used to differentiate between normal and abnormal breasts, highlighting the importance of focusing on these areas for cancer diagnosis. A novel region-based level-set method was developed for the automated segmentation of suspicious regions in breast thermograms in [21]. Subsequently, a three-layer feed-forward neural network was trained on higher-order statistical and GLCM-based texture features extracted from these segmented regions. Using a randomly selected subset of 50 thermograms with confirmed hot spots from the DMR-IR database, the model achieved an 89.4% accuracy in differentiating between malignant and benign breast tissue.

Additionally, several CAD systems have been proposed for breast cancer detection using thermal images, with a primary focus on feature selection techniques. A CAD system is presented in [22] employing Ant Colony Optimization (ACO) and Particle Swarm Optimization (PSO) for feature selection and SVM for classification, achieving accuracies of 94.29% and 97.14%, respectively, on the DMR-IR dataset. [23] introduced a CAD system utilizing PSO and Genetic Algorithms (GA) for feature selection and SVM for classification, reporting accuracies of 86.16% and 87.08% on a dataset of 336 thermal images after feature reduction. The CAD system proposed in [24] combining Extreme Learning Machines (ELM) and Optimized Directed Mutual Information (ODMI) for feature selection with various classifiers. Their system achieved a best accuracy of 98.49% on a dataset of 968 images while reducing the feature vector size by approximately 57%. [25] presented a CAD system employing Random Subspace Feature Selection (RSFS) and combinations of mRMR and GA with RSFS for feature selection and k-NN and SVM for classification. Their system achieved accuracies ranging from 77.41% to 87.83% on a dataset of 121 thermal images. A novel greedy search optimization method for feature selection within a Machine Learning (ML) framework introduced in [26]. They compared this approach to sequential backward, sequential forward, and exhaustive methods, finding that sequential backward selection was most effective for small datasets due to reduced redundancy and computational time with the accuracy of 88.57%.

Moreover, to classify images and extract relevant features, [27] integrated SVM with GA. The SVM was optimized through a 30-iteration process involving 140 participants in each iteration. Additionally, a GA with 100 iterations was employed to enhance SVM performance. The proposed method demonstrated high classification accuracy and an Area Under the Curve (AUC) of 97.91% when evaluated on the DMR-IR dataset. In addition, [28] proposed a novel image analysis and machine learning approach for breast cancer detection. Background noise was eliminated using the top-hat transform, followed by curve coefficient extraction through curve transform wrapping. Texture features derived from these coefficients were combined with geometric, statistical, and strength features to enhance classification accuracy. A comparative analysis of various machine learning methods revealed SVM as the superior classifier. Moreover, CNNs with Bayesian networks were used in [29] to improve breast cancer detection. Bayesian networks were introduced due to the black-box nature of Artificial Neural Networks (ANNs), which lack interpretability. This method was crucial for maintaining consistent probabilities and achieving full interpretability within the Bayesian network framework, as it is a probabilistic knowledge representation method. The CNNs and Bayesian networks were trained, evaluated, and integrated into a system, resulting in an improved diagnostic expert system. A model accuracy of up to 95% was achieved.

In addition, deep learning methods are being explored to analyze breast thermograms, aiming to improve early detection accuracy and potentially complement traditional diagnostic methods. A multi-input classification model was proposed by [30] to categorize breast thermal images. This model incorporated multiple perspectives of thermal images and patient-specific clinical data. By employing Convolutional Neural Networks (CNN), the model processed front, left (L90), and right (R90) view images simultaneously. The integration of diverse image angles with clinical information enhanced classification accuracy, as demonstrated by a 97% accuracy rate on the DMR-IR dataset. The study further highlighted the significant contribution of side view images in improving classification performance and the potential of clinical data for patient identification. In addition, [31] explored the optimization of CNN architectures using two biomimetic algorithms. Their primary objective was to determine the optimal structure for the fully connected layer to improve classification accuracy. The study revealed that GA surpassed PSO in refining model performance. Both optimization techniques outperformed traditional methods and exhibited adaptability to different network configurations. Besides, [32] introduces a novel breast segmentation approach that integrates curvature function (k) and gradient vector flow. Subsequently, a CNN was developed to classify the segmented breast images. The primary objective of this research was to evaluate the performance of CNN against traditional classification methods. To this end, each breast image was meticulously characterized by its shape, color, texture, and laterality (left or right). The resulting dataset was employed to train the CNN model and conduct comparative analyses with three established classifiers: random forest, multilayer perceptron, and Bayesian network. Experimental results demonstrate that the CNN model outperforms these conventional methods in breast image classification. Moreover, [33] employed PyTorch to preprocess and resize breast thermal images to a standardized 640x640 pixel format, excluding low-quality images. A CNN-based approach was adopted to detect breast cancer across five image views. Independent CNN models were developed and trained for each view, with their outputs subsequently combined to train a final neural network. This ensemble incorporated convolutional layers featuring 32, 64, and 128 channels, along with stride-2 and 2x2 pooling operations to reduce image dimensions to a probabilistic 1x2 matrix. Integrating clinical data into the CNN further enhanced performance, resulting in an improved accuracy of 93.80%. A pre-trained TransUNet network was first used to extract ROI areas from the DMR-IR dataset in [34]. The processed images were then inputted into four models, including EfficientNetB7 and VGG16, for classification. Accuracy rates of 98.39%, 97.26%, 77.53%, and 97.81% were achieved for EfficientNet-B7, ResNet-50, Visual Geometry Group (VGG)-16, and DenseNet-201, respectively. Additionally, [35] compared the performance of Inception V3, Inception V4, and a modified Inception V4 (MV4) model for classifying breast thermal images. Based on 467 experiments, all three models demonstrated classification capabilities with varying strengths. While Inception V3 excelled in grayscale image accuracy, both Inception V4 and MV4 exhibited strong performance with color images. Notably, the accuracy of the MV4 model improved with extended training time.

### 1.3. Contributions

The study’s significant contributions and key points can be outlined as follows:

- Unlike previous literature that has given less attention to nonlinear analysis of breast thermal images, this study applies various essential complexity measures such as fractal dimension, Lyapunov exponent, entropy, and correlation dimension. These measures capture different aspects of the chaotic characteristics of cancerous tumors.
- Texture features based on GLCM are employed to extract information from the images and calculate the characteristics of breast thermograms. Parameters such as contrast, correlation, energy, homogeneity, and diameter reflect aspects such as coarseness, linear dependency, textural uniformity, and pixel distribution within the image. Additionally, this paper introduces a combination of nonlinear and texture features to provide a comprehensive analysis of thermal images.
- A metaheuristic multi-objective optimization approach, NSGA III, is utilized to simultaneously minimize the objective function and the number of selected features and the results are evaluated in comparison to other metaheuristic optimization techniques, including GA, PSO, and Differential Evolution (DE) algorithms.
- The study employs k-fold cross-validation to mitigate the sensitivity of classification accuracy to training and testing datasets. It also explores diverse machine learning methods for effectively detecting breast cancer from digital thermography images.

**Fig 2:**
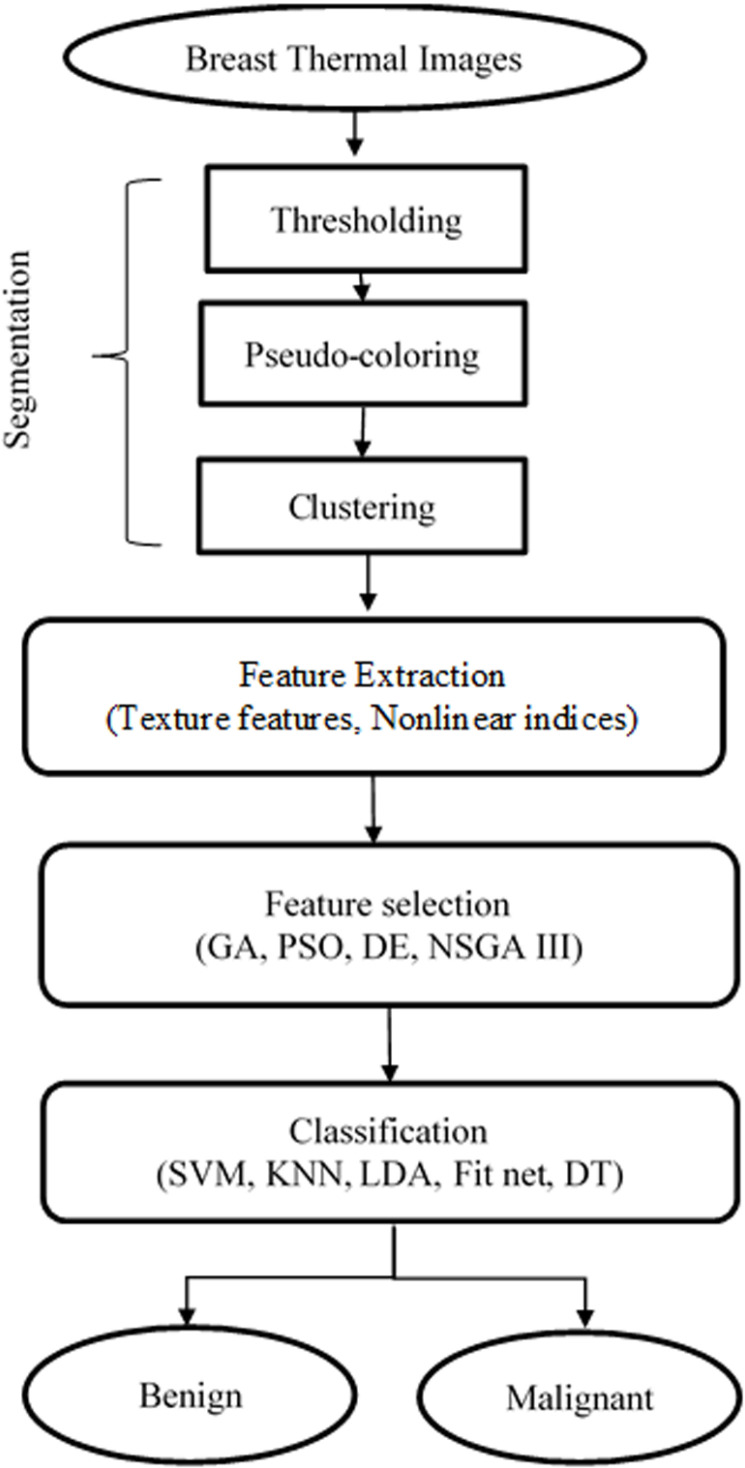
Flowchart of the presented approach.

Fig 2 illustrates the overall diagram of the proposed algorithm designed for diagnosing breast cancer from digital thermograms. Detailed explanations of each component are provided in the subsequent sections.

### 1.4. Paper organization

The structure of the remainder of this work is outlined as follows. Section 2 describes the input dataset of DMR-IR breast thermograms. In Section 3, the various stages of the proposed CAD system are detailed, including pre-processing, feature extraction, feature selection, classification techniques, and the classification function. Experimental results and performance evaluation are presented in Section 4. Section 5 compares the proposed method with other related works, while Section 6 conclude the obtained results.

## 2. The dataset

**Fig 3:**
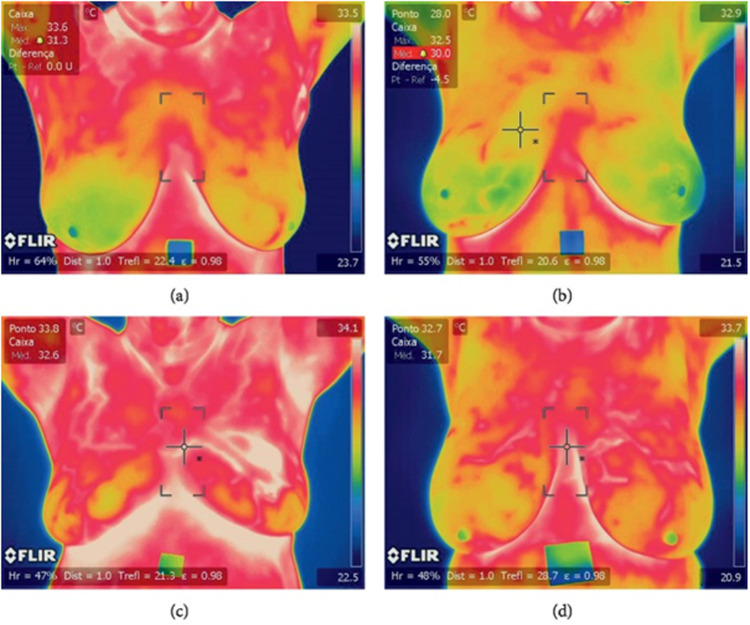
Breast theromographic images: (a, b): Healthy, (c, d): Sick [13,32].

The proposed method was evaluated using a benchmark dataset known as DMR-IR [13], which was compiled using Infrared (IR) images obtained from the Hospital of Universidade Federal Fluminense (UFF) University. Ethical approval was granted for the public release of this database, requiring patient consent for inclusion. Our study utilized 400 frontal thermogram images sourced from this database, captured using an FLIR SC-620 IR camera at a resolution of 640 × 480 pixels. For example, some of the achieved dataset images are demonstrated in Fig 3.

Each individual undergoes imaging in five distinct positions as part of the static protocol, namely Front, Right Lateral 45°, Right Lateral 90°, Left Lateral 45°, and Left Lateral 90°. Besides, 20 sequential images are captured in the front position, along with additional images taken from the Right Lateral 90° and Left Lateral 90° positions as part of the dynamic protocol. This comprehensive imaging approach ensures a thorough assessment across various angles and conditions for each individual.

### 2.1. Ethics statement

This study utilized the DMR-IR database, a publicly available and anonymized dataset. As the data were previously collected and fully anonymized, this secondary analysis did not involve direct interaction with human subjects or access to identifiable personal information. Consequently, no additional ethical approval was required for this research.

## 3. Proposed method

This section introduces a novel approach for distinguishing between benign and malignant tumors in IR breast images. The methodology encompasses several key stages: image segmentation involving thresholding, pseudo-coloring, and clustering; feature extraction incorporating texture and chaotic indices; feature selection utilizing different meta-heuristic algorithms; and classification employing various machine learning techniques based on the k-fold cross-validation method.

### 3.1. Segmentation

Assessing the malignancy or benignity of an IR image requires segmenting its different sections to isolate and extract the Region of Interest (ROI) from the thermograms. Given the specific color representation of different temperatures in infrared images, each section of the image corresponds to a distinct color indicative of a particular temperature. Analyzing features within the hottest regions of the thermograms enables the detection of abnormalities [36].

For this purpose, the grayscale breast thermographic images from the DMR-IR dataset were first thresholded using Otsu’s method. This method was employed for image thresholding in this study due to its simplicity, computational efficiency, and automated threshold selection based on histogram analysis. Widely used in image processing, Otsu’s method calculates an intensity threshold to divide pixels into two distinct classes: foreground and background by minimizing intra-class variance or maximizing inter-class variance. This approach is particularly effective for segmenting thermograms with distinct temperature differences between regions of interest and the background. As a non-parametric technique, Otsu’s method requires no prior assumptions about data distribution, making it suitable for diverse thermographic datasets. Compared to alternative techniques like manual, adaptive, iterative, k-means clustering, and entropy-based methods, Otsu’s method offers superior stability in segmentation results, eliminates the need for manual parameter adjustment, and benefits from widespread validation and adoption in medical image processing. Its algorithmic equivalence to globally optimizing a k-means clustering algorithm applied to the image intensity histogram further highlights its robustness for precise delineation of object boundaries [37].

Subsequently, the Hue, Saturation, Intensity (HIS) color space was employed for pseudo-coloring the grayscale images. Pseudo-coloring is a technique that transforms grayscale thermal images into colorized visuals by mapping intensity values to a predefined color spectrum. This process assigns distinct colors to temperature ranges, with hotter areas often appearing in red and yellow and cooler areas in blue and turquoise, visually highlighting regions of abnormal heat distribution. By enhancing the visibility of subtle intensity differences, pseudo-coloring improves input quality for automated feature extraction algorithms and supports healthcare professionals in identifying potential malignancies. Additionally, the human eye’s heightened sensitivity to color differences makes pseudo-coloring effective for emphasizing specific image attributes, aiding in clinical interpretation without advanced tools. The HSI color space is often used for this purpose, with hue, saturation, and intensity selected as coordinate axes. More details about this algorithm are provided in [38,39].

Finally, the Fuzzy C-Means (FCM) clustering algorithm was utilized to separate the various clusters within the image and extract its ROI. This algorithm was chosen in the current study due to its ability to handle ambiguity and overlapping clusters, crucial for analyzing breast thermograms [40]. Unlike strict clustering methods like k-means, FCM assigns membership values to pixels, reflecting gradual transitions in temperature. This flexibility is essential for capturing subtle intensity variations in thermograms, aiding in tumor detection and segmentation [41]. Various similarity measures such as distance, connectivity, and intensity are used to identify groups in different datasets and applications. In this research, feature values are leveraged to classify images into four groups: white matter, grey matter, Cerebrospinal Fluid (CSF), and abnormal tumor regions. While membership functions from FCM do not directly reveal real data distributions or facilitate fuzzy pattern recognition, they are constructed using available data. To achieve this, an appropriate clustering algorithm is essential for grouping data based on similarity or dissimilarity measures like distance. The C-means clustering algorithm, a simple unsupervised learning method suitable when the number of clusters is known, is employed in this work. The FCM method for image segmentation involves specific steps outlined in [42].

**Step 1:** Assign a number (k ) to denote the clusters.

**Step 2:** Arbitrarily select k vectors $c_{1},c_{2}\ldots c_{k}$ from the learning data as the initial centers of clusters.

**Step 3:** Employ the Euclidean distance measure to assign each vector $x_{l}=[x_{l1},x_{l2},...,x_{\ln}{]}^{T}$ (where the dimension of input vectors is denoted by n) to the cluster with the closest center $c_{i}$:


$$\mathrm{\backslash [}\left\|x_{l}-c_{i}\right\|=\underset{j}{\min}\left\|x_{l}-c_{j}\right\|\mathrm{\backslash ]}$$ (1)

**Step 4:** Recalculate the estimated cluster centers $c_{i}$. Let $c_{i}=[c_{i1},c_{i2},...,c_{in}{]}^{T}$ be the vectors assigned to cluster *i*, and *m* be a fuzziness exponent. The new cluster center ***c***_*im*_\m is obtained as:


$$\mathrm{\backslash [}c_{im}=\frac{\sum x_{li}\in Cluster(lx\lim)}{N_{i}}\mathrm{\backslash ]}$$ (2)

where the number of vectors in the ***i***_*th*_ cluster is denoted by ***N***_*i*_.

**Step 5:** If all of the cluster centers $\left(c_{i},i=1,2,\ldots ,k\right)$ do not change in Step 4, terminate the algorithm; otherwise, return to Step 3.

### 3.2. Feature extraction

As mentioned previously, this study concentrates on two categories of extracted indices: complexity measures or nonlinear indices, and texture features.

#### 3.2.1. Texture features.

Texture features or statistical analyses are essential tools for quantifying characteristics of breast thermograms and extracting pertinent information from images. These tools are instrumental in addressing specific computational tasks relevant to distinct applications. One commonly employed statistical approach for describing textural features is the GLCM, which effectively captures spatial relationships between pixels with varying gray levels [43,44].

A range of GLCM descriptors, including Autocorrelation, Correlation, Energy, Cluster Prominence, Cluster Shade, Dissimilarity, Maximum Probability, Variance, Entropy, Sum Average, Sum Variance, Sum Entropy, Difference Variance, Difference Entropy, Information Measure of Correlation1, Information Measure of Correlation2, Inverse Difference Normalized (IDN), Inverse Difference Moment Normalized, Homogeneity, Contrast, and Inverse Difference Moment (IDM) [45], are utilized alongside the Discrete Wavelet Transform (DWT) in this study for the extraction of statistical, and textural features from the thermal images and thus, providing valuable insights into the analyzed data.

#### 3.2.2. Chaotic indices.

Chaotic time series offer valuable tools for developing procedures to assess nonlinear systems. In the case of biomedical image processing, leveraging nonlinear analysis and chaotic indices can effectively characterize complex patterns within the data [44]. This study utilizes techniques such as FD, LE, KSE, and CD to offer robust tools for analyzing complex and dynamic phenomena evident in biomedical images. These methods contribute to enhancing diagnostic approaches within the field.

*Fractal dimension*: The concept of FD relates to a geometric representation across multiple dimensions, where the inherent structure of the original image remains consistent irrespective of scale. This quality of self-similarity seen in fractal images suggests that every subsection tolerates resemblance to the original image. The “partial dimensions” concept can be effectively described using the FD, with the Box-Counting Method (BCM) being a popular approach for FD estimation in the literature. The BCM involves dividing the image into square boxes of similar sizes and counting the number of boxes that cover parts of the image. By repeating this process with smaller boxes, the numbers of containing boxes are counted at each step. The FD of the pattern is then determined by plotting the logarithm of the counted box numbers against the logarithm of the magnification index for each partitioning step and calculating the slope of the best-fitting line. This slope represents the FD (*D*) value, providing insight into the complexity and structure of the strange attractor [40].


$$\mathrm{\backslash [}a\propto \frac{1}{S^{D}}\mathrm{\backslash ]}$$ (3)

where


$$\mathrm{\backslash [}D=\frac{\log(a)}{\log(\frac{1}{s})}\mathrm{\backslash ]}$$ (4)

Lyapunov exponent: LE serves as a metric for quantifying how rapidly two trajectories diverge when they originate from nearly identical initial conditions within a state space. This sensitivity to initial conditions is a fundamental characteristic of chaotic systems. By calculating the LE, one can gauge the degree of chaos present within the system. In this study, we utilize the same methodology for computing LE as outlined in [46]. This approach involves reconstructing a pseudo Phase Space (PS) through a Time-Delay Embedding (TDE) technique applied to the breast thermal image time series. Subsequently, the Lyapunov exponents are determined using the Jacobian method. In this study, the largest Lyapunov exponent (LLE) is regarded as an index.

*K-S entropy*: Entropy, defined as the logarithm of the number of microstates of a dynamic system [47], reflects the system’s level of irregularity. KS entropy is a modified version of Approximate Entropy (ApEn) and serves as a measure of complexity in dynamic systems. A low ApEn value typically indicates high predictability and low complexity within a time series. KS entropy, in contrast, demonstrates a high degree of independence from the length of historical data and maintains a consistent value compared to ApEn. Additionally, the algorithm for calculating KS entropy is simpler than that for ApEn, resulting in reduced computation time. The equation representing KS entropy for a symbol of length N is expressed as Equation (5).


$$KSEn(m.r)=\lim_{N\to \infty }\{-ln\frac{C^{m+1}(r)}{C^{m}(r)}\}$$ (5)

$C^{m}(r)$represents the probability of similarity between two sequences within m points (based on distance), while $C^{m+1}(r)$ denotes the probability of similarity within m+1 points. When N is constrained to a specific value, the entropy value of the estimated sample can be expressed using Equation (6).


$$\mathrm{\backslash [}KSEn(m.r)=-ln\frac{C^{m+1}(r)}{C^{m}(r)}\mathrm{\backslash ]}$$ (6)

In this equation, m refers to the length of the sequence being examined, and r represents the tolerance level used to determine the similarity between sequences [46].

Correlation dimension: One method for assessing the chaotic nature of a signal is by calculating the correlation dimension. Several methods exist for computing the correlation dimension, such as Grossberger’s method, Thickens’ method, Chord’s method, and Ellner’s method. In Grossberger’s method, the first step involves determining the distance between each pair of points within the phase space. The correlation sum for each radius r can be calculated as the sum, over all points on the attractor, of the count of points within radius r, normalized by the total number of points N in the attractor as (7).


$$C(r)=\frac{2}{N(N-1)}\sum_{i.j}^{N}\theta (r-\left|x_{i}-x_{j}\right|)$$ (7)

In Equation (7), the norm denotes the Euclidean distance between points, where r represents the neighborhood radius, N is the total number of points, and θ is the Heaviside function. The term preceding the summation represents the reciprocal of the total number of possible vector pairs that could be formed. Therefore, we can interpret the correlation sum as the probability that a randomly selected vector pair will be closer than r. After calculating the correlation sum with different r values, the correlation dimension is determined by the slope of a log-log plot of the correlation sum against the radius r [48].

### 3.3. Feature selection with meta-heuristic algorithms

Metaheuristic techniques are considered highly effective for solving optimization problems, particularly in feature selection tasks. They excel at efficiently navigating complex scenarios to find optimal solutions.

*Genetic algorithm* GAs are versatile techniques that can effectively address various optimization problems, including those with constraints and without constraints. By employing iterative processes such as mutation, crossover, and selection, GAs generate optimal solutions from the initial population [49].

*Particle swarm optimization* This method mimics the behavior of social animals to achieve optimization goals. By continuously modifying particle positions and velocities, PSO strives to minimize optimization challenges and attain optimal solutions [50].

#### Differential evolution.

 this method overcomes a significant drawback of GA, particularly their struggle with local search. Unlike GA, which selects parents based on their fitness, DE offers equal opportunities for all solutions to be chosen. After undergoing mutation and crossover, a new solution is assessed against the previous one and substituted if it shows better performance [51].

#### NSGA-III algorithm.

 The fundamental structure of this approach closely resembles the well-known NSGA-II [52], albeit with significant alterations in its selection methodology. Main difference can be summarized as follows:

1. **Initialization**: NSGA-III starts by defining reference points and generating an initial population of size N.
2. **Iterative Process**: At each generation (t), the current parent population ($P_{t}$) produces an offspring population ($Q_{t}$) using random selection, SBX crossover, and polynomial mutation [53]. Both $P_{t}$ and $Q_{t}$ have N members, forming a merged population ($R_{t}$) of size 2N.
3. **Selection**: Non-dominated sorting categorizes $R_{t}$ into non-domination levels ($F_{1}$, $F_{2}$, etc.), and a new population ($S_{t}$) is created by selecting members from different levels until reaching N or exceeding it.
4. **Modified Selection**: NSGA-III alters its selection approach by analyzing members in $S_{t}$ based on normalized objective values and reference points. A niche-preservation operation ensures diversity and optimal selection from the previous fronts.

This efficient process maintains diversity and improves selection efficiency, enhancing NSGA-III’s performance in optimization tasks. For detailed insights, refer to [54].

### 3.4. Cross-validation

To mitigate the over-fitting effect and enhance classifier reliability, the k-fold cross-validation method is utilized [55]. Using K=10 as an example, the dataset is divided into ten random combinations of training and test sets. Each subsample is of equal size, with nine used for training and one for testing in each iteration. This process is repeated ten times, ensuring all subsamples are utilized for both training and testing stages.

### 3.5. Classification

To assess the effectiveness of selected computational features in distinguishing breast thermograms, five different machine learning classifiers, namely KNN, SVM, LDA, Pat net, and Fit net, are utilized.

*K-nearest neighbors* The KNN algorithm is acknowledged as a simple yet effective classification method that works by categorizing an input feature vector according to its proximity to the k nearest training vectors, identified using a suitable distance metric. The vector is then assigned to the class with the highest popularity among its nearest neighbors. KNN classification is a traditional nonparametric supervised technique renowned for its favorable accuracy, especially when the optimal parameter k is chosen considerately [56].

*Support vector machine* SVM is a robust tool used for classifying various image sets. It functions by separating data points into separate categories using a hyperplane, which maximizes the margin between the categories. The Radial Basis Function (RBF) kernel is frequently employed in SVM due to its flexibility. Equation (8) delineates the decision function linked with the RBF kernel:


$$\mathrm{\backslash [}C(x)=Sgn\left[\sum_{i=1}^{Nv}y_{i\in i.}K(s_{i},x)+b\right]\mathrm{\backslash ]}$$ (8)

In Eq. (8), $y_{i\in i}$ is the Lagrange coefficient, $s_{i}$, $i=1...N_{v}$ represents the separating plane and denotes the bias term and the number of support vectors is denoted by $N_{v}$. Additionally, Eq. (9) as a Gaussian radial basis function is applied in SVM to handle nonlinear data and defines the decision function [57].

$$\mathrm{\backslash [}K(u,v)=\exp(\frac{-{\left\|u-v\right\|}^{2}}{2\sigma^{2}})\mathrm{\backslash ]}$$ (9)

In which, σ is the variance of the selected hyper parameter and $\begin{array}{c} ||u-v|{|}^{2}\; \end{array}$ denotes the Euclidean distance between these two points.

*Linear discriminant analysis*: LDA is a probabilistic method that operates under the assumption that the probability of a sample belonging to a specific class follows a multivariate Gaussian distribution. LDA calculates this probability based on a formula known as the classification law. From a geometric perspective, this involves defining decision boundaries that partition the v-dimensional hyperspace into regions corresponding to different classes. Notably, the separating hypersurfaces in the multidimensional space are linear, hence it is known as LDA [58].

*Pattern recognition network* In the Pat net approach, inputs are categorized into target classes through the training of an artificial neural network. For pattern recognition networks in Pat net, the target data must consist of zero-valued vectors with only one element set to 1, representing the target class [55].

*Fitting neural network* In Fit net methodology, the training process adapts performance based on a specific input set and generates corresponding target outputs. Once the network structure, including desired hidden layers, is established, training is conducted using a designated training dataset. As the neural network learns from the data, it constructs a representation of the input-output relationship [55].

### 3.6. Performance evaluation

To assess the effectiveness of the proposed approach, various statistical metrics are employed, such as True Positive (TP), True Negative (TN), False Negative (FN), and False Positive (FP). These metrics play a crucial role in calculating important statistical functions. Sensitivity, specificity, and accuracy are fundamental statistical metrics defined as follows (see Equation 10) [59].


$$\mathrm{\backslash [}\begin{array}{c} ACC=\left(\sum_{i=1}^{N}\frac{TP+TN}{TP+FP+TN+FN}\right)\mathrm{.100}\\ \%/N\\ SEN=\left(\sum_{i=1}^{N}\frac{TP}{TP+FN}\right)\mathrm{.100}\\ \%/N\\ SPE=\left(\sum_{i=1}^{N}\frac{TN}{FP+TN}\right)\mathrm{.100}\\ \%/N \end{array}\mathrm{\backslash ]}$$ (10)

## 4. Experimental results and discussion

The breast cancer diagnosis method proposed in this study is applied to DMR-IR thermographic images. All simulations are performed on a system featuring a 2.6 GHz Core i7-10750 CPU using MATLAB R2022a (MathWorks Inc.).

**Fig. 4:**
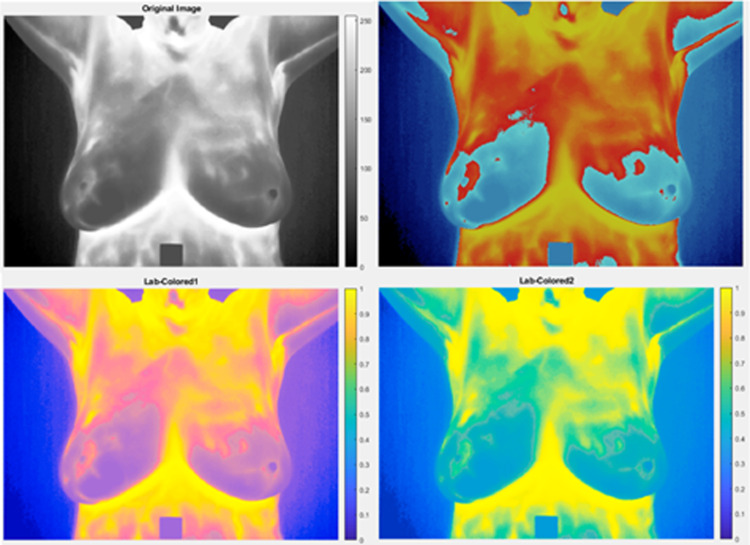
Results of pseudo-coloring algorithms for a normal thermogram of an individual: original gray-level image, and its pseudo-colorized images in the HSI color space under various conditions.

**Fig. 5:**
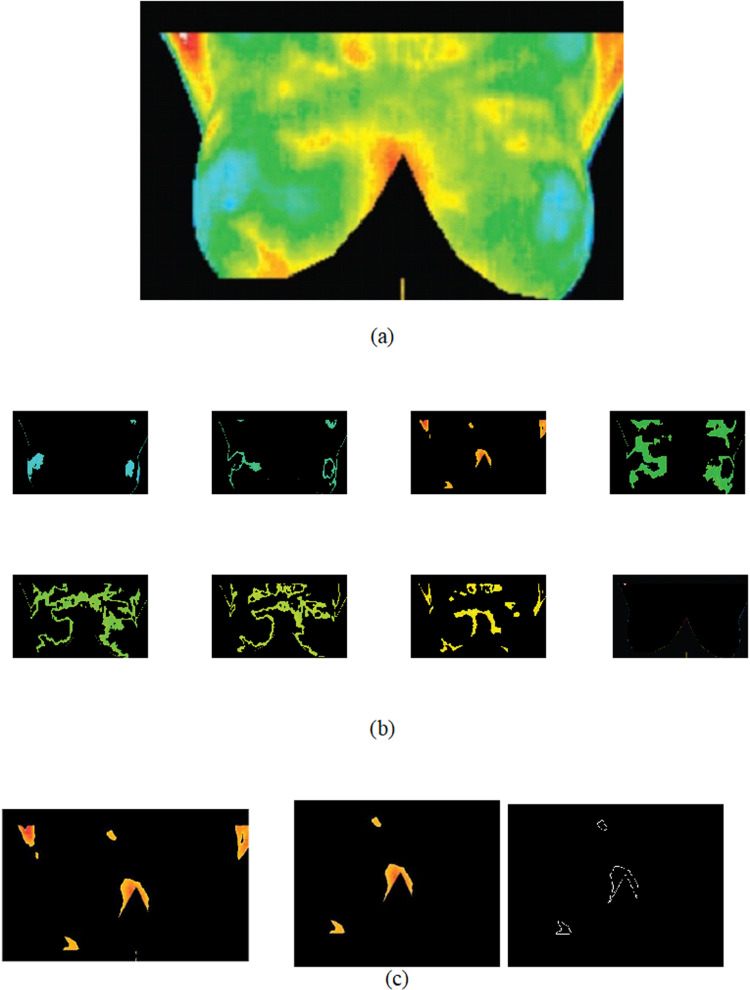
Breast thermograms clustering using FCM method. (a) Pseudo-colorized image, (b) clustered image applying the FCM method, (c) extracted hottest region after removing the axilla and sternal and the final binary image.

The initial step in distinguishing masses from the original image involved pseudo-coloring the existing grayscale image. The images in the DMR-IR database are in the form of a thermal matrix and include images captured from various angles of the breast. Initially, the grayscale images were transformed into color images using the HSI method, and the resulting conversion output can be observed in Fig 4. This pseudo-colorized image, depicting various temperature ranges, can be utilized for different preferences or analyses. In addition, the Fig 5 illustrate an IR breast thermographic pseudo-colorized image, color clustering using the FCM method, extraction of its hottest region, the hottest region after removing the axilla and sternal regions and the final binary image. The binary image can be utilized for the extraction of nonlinear features.

In the following step, complex indices introduced in the study are computed for the images in the DMR-IR dataset. Table 1 presents the averages and standard deviations of these indices. The a ± b is the selected symbol for this presentation in which, a denotes the averages and b presents the standard deviations. Table 2 presents the averages and standard deviations of texture features for both benign and malignant cases, similar to the preceding table.

**Table 1:** The mean ± std. of chaotic features for all benign and malignant cases.

Features	Benign	Malignant
FD	1.121±0.1735	1.5168±0.1919
LLE	1.8923±0.1357	2.1109±0.1608
KSE	2.3412±0.1132	2.5561±0.1361
CD	0.9433±0.0439	1.2155±0.04911

**Table 2:** The mean ± std. of texture features of benign and malignant cases for all breast thermograms.

Features	Benign	Malignant
Autocorrelation	22.0078±2.78	23.3823±2.31
Contrast	0.4573±0.08	0.4123±0.07
Correlation	1.0239±0.03	0.9933±0.02
Cluster Prominence	311.0777±122.17	388.7629±154.99
Cluster Shade	−23.8921±10.6	−41.2004±18.33
Dissimilarity	0.0618±0.02	0.1061±0.03
Energy	0.6044±0.15	0.6671±0.15
Entropy	1.3116±0.38	1.6169±0.39
Homogeneity	0.7209±0.02	0.6911±0.02
Maximum probability	0.2399±0.1	0.4081±0.13
Variance	37.1209±3.18	38.0133±5.11
Sum average	11.1376±0.77	12.1099±0.94
Sum variance	124.2369±20.33	126.5858±22.37
Sum entropy	1.3374±0.19	1.5407±0.29
Difference variance	0.3121±0.09	0.3718±0.09
Difference entropy	0.5104±0.13	0.5411±0.21
Information measure of correlation1	−0.7126±0.05	−0.7923±0.04
Informaiton measure of correlation2	1.1387±0.05	1.4355±0.06
INN	0.8169±0.01	0.9971±0.01
IDN	0.7691±0.01	0.8156±0.01

Tables 1 and 2 demonstrate significant variations in specific features between benign and malignant breast thermograms. However, some features exhibit minimal distinctions. This highlights the necessity of employing feature selection techniques to improve recognition accuracy. Feature selection achieves this by discarding irrelevant data and reducing the dimensionality of the feature set. Following the extraction of diverse features from breast thermograms, meta-heuristic algorithms are employed for feature selection. GA, PSO, DE, and NSGA III are utilized in this process. These algorithms identify the most highly correlated features by incorporating both complexity and texture indices extracted from the images. Table 3 presents an overview of the design parameters employed within these feature selection methods to facilitate equitable comparison.

**Table 3:** The design parameters for feature selection methods.

Feature selection algorithms	Parameters values
GA	• Maximum number of iterations = 20; • Population size (npop) = 30; • Crossover percentage (pc) = 0.8; • Number of off springs (Parents) = 2*round (pc*nPop/2); • Mutation percentage (pm) = 0.3; • Number of mutants (nm) = round(pm*nPop); • Mutation rate (mu) = 0.02; • Selection pressure = 8;
PSO	• Maximum number of iterations = 20; • Population size (swarm size) = 20; • Inertia weight = 1; • Inertia weight damping ratio = 0.99; • Personal learning coefficient = 2; • Global learning coefficient = 2;
DE	• Maximum number of iterations = 20; • Population size (npop) = 20; • Lower Bound of Scaling Factor (beta_min)=0.2; • Upper Bound of Scaling Factor (beta_max)=0.8; • Crossover Probability (pCR)=0.2;
NSGA III	• Maximum number of iterations = 20; • Population size (npop) = 30; • Crossover percentage (pCrossover) = 0.7; • Mutation Percentage (pMutation)=0.4; • Number of Generations (T): = round(pMutation*nPop); • Mutation rate = 0.1;

Besides, Table 4 presents the results of the optimization process, including the optimal objective function values achieved, the number of features selected, and the corresponding implementation times. Among these techniques, NSGA III, with its multi-objective optimization capability, strikes a balance between minimizing the objective function and selecting an appropriate feature subset. While this method incurs a longer runtime compared to other approaches, its superior performance justifies this trade-off, particularly within the context of offline implementations. The convergence plot (Fig 6) visually depicts the convergence behavior of the objective function value with increasing iterations, ultimately reaching the minimum value.

**Table 4:** The final values of the cost function, the selected features number, and the implementation time for the introduced meta-heuristic algorithms.

	Metaheuristic algorithms			
	GA	PSO	DE	NSGA III
The ultimate values of the objective function	0.0601	0.0782	0.0809	0.0441
Number of selected feature	9	7	9	12
Implementation time	2451sec	1176sec	1201sec	4230sec

**Fig. 6:**
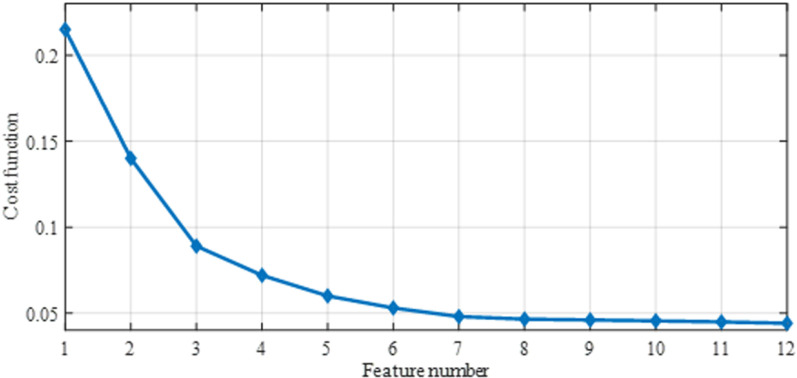
The objective function amounts versus the selected feature number for the NSGA III method.

As observed, there is not a substantial difference in the value of the objective function after selecting eight features. Therefore, eight indices are chosen for classification, considering the balance between minimizing the cost function and managing computational complexity. These features, identified by NSGA III, act as the input for the classifiers. Seventy percent of the breast thermogram data was used to train the classifiers, while the remaining 30% was reserved for testing. Details on the specific classification models can be found in Table 5.

**Table 5:** Further information regarding the designed classifiers.

Classifier	Parameters values
SVM	• ‘Kernel function’ is ‘gaussian’
KNN	• Number of nearest neighbors = 5
LDA	• ‘DiscrimType’ is ‘pseudoquadratic’
Pat net	• Activation function: Levenberg-Marquardt
Fit net	• Activation function: Levenberg-Marquardt

The more correlated features identified by NSGA III are used as input for the classification stage. Here, five established classification algorithms are employed: KNN, SVM, LDA, Pat net, and Fit net. To assess the effectiveness of these classifiers, a 10-fold cross-validation methodology is implemented. This process involves randomly dividing the dataset images into ten equal-sized folds. During each iteration, nine folds are used to train the models, and the remaining fold is used for testing. This cycle is repeated ten times, ensuring all data points contribute to both training and testing phases.

**Fig. 7:**
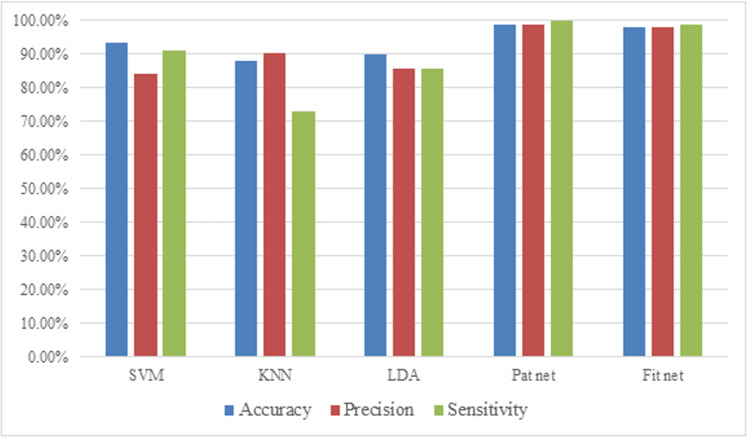
The bar graph depicting the statistical metrics of the designed classifiers applying 10-fold cross-validation method.

Table 5 details the specific parameter settings used for each of the five employed classifiers. These parameters are critical for optimizing the models’ performance and achieving accurate classification of breast thermograms. To comprehensively assess the effectiveness of these classifiers in differentiating between benign and malignant tumors, various performance metrics are utilized. These include accuracy, sensitivity and specificity. The results, represented in Table 6 and Fig 7, highlight the outstanding performance of the proposed algorithm in distinguishing between malignant and benign breast tumors. Notably, Pat net classifier achieved high accuracy, aligning well with expectations. These findings further confirm the efficacy of the proposed method in facilitating automated, effective, and swift detection of breast tumors.

**Table 6:** The achieved outcomes regarding the statistical metrics of the designed classifiers.

	SVM	KNN	LDA	Pat net	Fit net
Accuracy	93.33%	88.12%	90.01%	98.65%	97.97%
Precision	84%	90.12%	85.71%	98.78%	98.06%
Sensitivity	90.91%	72.86%	85.71%	99.79%	98.83%

### 4.1. Evaluation of the proposed method

**Fig. 8:**
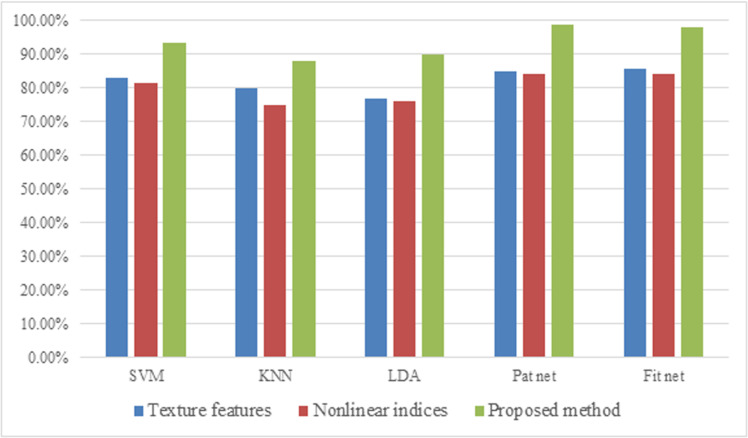
The graph bar of the accuracy comparison for three types of feature sets applying 10-fold cross-validation method.

As previously mentioned, this study builds on the idea of combining texture analysis with non-linear methods to extract features from breast thermal images. To show how well this approach works, an experiment is performed. Different classifiers were employed, utilizing both texture-based and non-linear features independently for the classification of health status within the thermograms. Similar to the previous experiment, 10-fold cross-validation was applied to eliminate the over-fitting effect and enhance the reliability of the classifiers. The accuracy achieved for these classifications is presented in Table 7. Furthermore, a comparison between the evaluation metrics of the selected classifiers for various extracted feature types and the proposed method is illustrated in Fig 8.

**Table 7:** Accuracy related to different feature domains on different classifications.

	SVM	KNN	LDA	Pat net	Fit net
Texture features	83.09%	79.91%	76.65%	85.04%	85.76%
Nonlinear indices	81.55%	75.08%	75.95%	83.94%	84.09%
Proposed method	93.33%	88.12%	90.01%	98.65%	97.97%

Table 7 and Fig 8 showcase the effectiveness of the proposed method, which extracts features by combining texture analysis with non-linear methods, for cancer diagnosing trough breast thermograms. When using the Pat net classifier, which achieved the best results, accuracy reached approximately 85% for texture-based features and 84% for features derived from chaotic characteristics. These results recommend that using only one type of feature is not sufficient for high classification accuracy, highlighting the benefit of combining them. Similar trends are observed with other classifiers. Notably, the features chosen by the NSGA III algorithm include representatives from both texture and non-linear categories, further emphasizing the importance of utilizing them together.

### 4.2. Comparison with other studies

In order to evaluate the effectiveness of the proposed method in classifying benign and malignant tumors within breast thermal images, a comparison was conducted between its achieved results and those of other techniques employed in prior research utilizing the same dataset. Table 8 summarizes various studies that were performed on a range of datasets, with a particular focus on DMR-IR. These studies implemented diverse feature extraction methods, feature selection approaches and machine learning algorithms for breast cancer detection. The explored approaches encompassed Bayes network, Naïve Bayes, SVM, DT, Multilayer Perceptron (MLP), ELMs, KNN, ANN, RBF, and various deep neural network methods such as CNNs, multi-input CNNs, CNNs with Bayes optimization, transfer learning CNNs, and Mask R-CNN. As illustrated in Table 8, the proposed method by this paper outperforms these existing approaches in the diagnosis of breast cancer from thermal images.

**Table 8:** A comparison between accuracies of the present method and the other published results used the same database (DMR-IR).

Accuracy	Approaches	Dataset	Publication Year	Ref
97.14%	PSO, SVM	DMR-IR	2019	[22]
72.18%	SR-based thermal analysis system	DMR-IR, DBT-TU-JU	2019	[20]
97%	Multi-input CNN	DMR-IR	2019	[30]
87.08%	PSO and GA, SVM	DMR-IR	2019	[23]
98.49%	ELM, ODMI	DMR-IR	2020	[24]
94.3%	AlexNet	DMR	2020	[60]
95.6%	DVLF, FBN	DMR-IR	2021	[61]
97.18%	GA, SVM	DMR-IR, UFPR	2021	[27]
93.80%	CNN	DMR-IR	2022	[33]
98.36%	ResNet50, VGG16	DMR	2022	[34]
91.66%	Bio-inspired algorithms, CNN	DMR-IR	2022	[31]
95.00%	CNNs with Bayesian networks	DMR-IR	2023	[29]
97.1%	Mask R-CNN	DMR-IR	2023	[1]
88.57%	Greedy search optimization, ML	DMR-IR	2023	[26]
90.00%	Transfer learning, GAN neural networks	DMR-IR	2024	[62]
93.33% 88.12% 90.01% 98.65% 97.97%	Combination of texture and nonlinear features, selected by NSGA III, several classifier	DMR-IR	–	Proposed method

## 5. Conclusion

This study introduces a novel CAD system for breast cancer diagnosis using thermal imaging. The system integrates comprehensive feature extraction with a wide variety of features, optimized feature selection via multi-objective optimization approach, and versatile classification through various machine learning algorithms. Experimental results demonstrate significant improvements in detection accuracy, with the Pat Net classifier achieving the highest performance. The method is automated, efficient, and offers rapid processing for tumor diagnosis using breast thermograms. A noted limitation is the extended processing time required by the NSGA III feature selection method; however, this primarily affects offline stages and does not inhibit real-time image recognition. Future work will focus on optimizing processing times and exploring advanced algorithms to further enhance system accuracy and efficiency.
